# Cytotoxic profiling of *Hedera pastuchovii* in human cancer cell lines and its genoprotective effects on normal lymphocytes

**DOI:** 10.1038/s41598-026-61972-y

**Published:** 2026-08-12

**Authors:** Emran Habibi, Mohammad Shokrzadeh, Omid Abed Khojasteh, Hashem Gerey, Mohammad Hossein Hosseinzadeh, Satyajit D. Sarker, Lutfun Nahar

**Affiliations:** 1 Centre for Natural Products Discovery, School of Pharmacy and Biomolecular Sciences, Liverpool John Moores University, Byrom Street, Liverpool, L3 3AF UK; 2 Medicinal Plants Research Centre, Institute of Herbal Medicines and Metabolic Disorders, Mazandaran University of Medical Sciences, Sari, Iran; 3 Department of Toxicology and Pharmacology, Faculty of Pharmacy, Mazandaran University of Medical Sciences, Sari, Iran; 4 Department of Clinical Pharmacy, School of Pharmacy, Guilan University of Medical Sciences, Rasht, Iran; 5 Pharmaceutical Sciences Research Center, Hemoglobinopathy Institute, Mazandaran University of Medical Sciences, Sari, Iran; 6 Laboratory of Growth Regulators, Palacký University and Institute of Experimental Botany, The Czech Academy of Sciences, Šlechtitelů 27, 78371 Olomouc, Czech Republic

**Keywords:** *Hedera pastuchovii*, Triterpenoid saponins, Cytotoxicity, Apoptosis induction, Genoprotective effect, Biochemistry, Cancer, Chemical biology, Drug discovery, Plant sciences

## Abstract

The genus *Hedera* L. has long been recognized as a valuable source of anticancer agents, such as triterpenoid saponins. In contrast, *Hedera pastuchovii* Woronow remains largely unexplored. This study investigates the cytotoxic profiles and genoprotective potential of *H. pastuchovii* leaf extracts. Leaves were extracted with ethanol and fractionated into *n*-hexane (Hex-F), ethyl acetate (Eth-F), and methanolic (Met-F) fractions using vacuum liquid chromatography. Phenolic, flavonoid, and saponin contents were quantified. Cytotoxicity was evaluated against four human cancer cell lines: A549 (lung), MCF-7 (breast), HeLa (cervical), and SKOV3 (ovarian), using the MTT assay. Apoptosis was assessed by Annexin V/PI flow cytometry, and genotoxicity or genoprotective effects were measured by the micronucleus assay in human lymphocytes. Phytochemical analysis revealed a total saponin yield of 29.95%. The Met-F fraction exhibited the highest concentrations of total phenolics (77.93 mg GAE/g) and triterpenoids (138.62 mg UAE/g), as well as the most pronounced cytotoxic activity, significantly reducing cell viability across all tested cancer lines in a concentration-dependent manner. The IC_50_ values were 108.23, 150.70, 213.77, and 781.07 µg/mL for SKOV3, MCF-7, HeLa, and A549 cell lines, respectively. In comparison, the Hex-F and Eth-F demonstrated weak cytotoxicity (IC_50_ > 1000 µg/mL). Flow cytometry suggested apoptosis as the predominant mode of cell death induced by Met-F. The micronucleus assay showed that Met-F reduced cisplatin-induced micronuclei formation in lymphocytes in a dose-dependent manner, indicating measurable genoprotective activity. These findings provide preliminary evidence that *H. pastuchovii* leaf fractions, particularly Met-F, possess in vitro cytotoxic and genoprotective properties that warrant further investigation.

## Introduction

Cancer is a complex genetic disease marked by the uncontrolled growth of cells driven by multiple oncogenic mutations^1^. This continuous proliferation disrupts the body’s normal regulatory mechanisms, leading to resistance to apoptosis and the development of invasive and metastatic properties. Because of these characteristics, cancer cells are highly resilient, and effective management usually demands a combination of treatments including surgery, chemotherapy, radiotherapy, and targeted therapy^2^. Each year, around 10 million people die from cancer, making it the second leading cause of death worldwide after cardiovascular diseases^3^. Despite advances in diagnosis and treatment, the search for more effective and safer pharmacological agents for cancer therapy remains an urgent global priority.

According to GLOBOCAN 2022 data, lung cancer is the most diagnosed cancer globally and the leading cause of cancer-related deaths, accounting for over 1.8 million deaths worldwide^4^. Breast cancer ranks second in overall incidence, while being the most prevalent cancer among women and the second leading cause of cancer death in the female population. Cervical cancer remains a common malignancy in densely populated African and Asian countries^4^. Despite advancements in treatment options and the availability of preventive vaccinations, it continues to be a major cause of mortality in less developed and low-income countries. Finally, although ovarian cancer has a lower incidence rate, its relatively high mortality, particularly when diagnosed at a late stage, makes it a significant clinical challenge, especially among postmenopausal and older women^4^. These statistics emphasize the urgent need for new anticancer agents.

The cancer cell lines used in this study represent well-established in vitro models for major human malignancies. The A549 cell line, derived from lung adenocarcinoma in 1973, closely resembles respiratory epithelium and is widely used in lung cancer research^5^. The MCF-7 cell line, extensively utilized in breast cancer research, was first established in 1973 by Dr. Soule and his team at the Michigan Cancer Foundation. It originated from the pleural fluid of a 69-year-old patient diagnosed with metastatic breast carcinoma^6^. The HeLa cell line, the first immortalized human cancer model, was derived in 1951 from an aggressive cervical adenocarcinoma in a 30-year-old patient treated at Johns Hopkins Hospital. HeLa cells are the most extensively utilized human cell model in biomedical sciences and cancer research^7^. The SKOV3 cell line, derived from ovarian carcinoma, is a reliable model for ovarian cancer research^8,9^.

Medicinal plants and natural products have long been a valuable source of bioactive compounds for the discovery and development of new therapeutics. A well-known example is the isolation of paclitaxel from *Taxus brevifolia*, which revolutionized cancer treatment^10,11^.

**Fig. 1:**
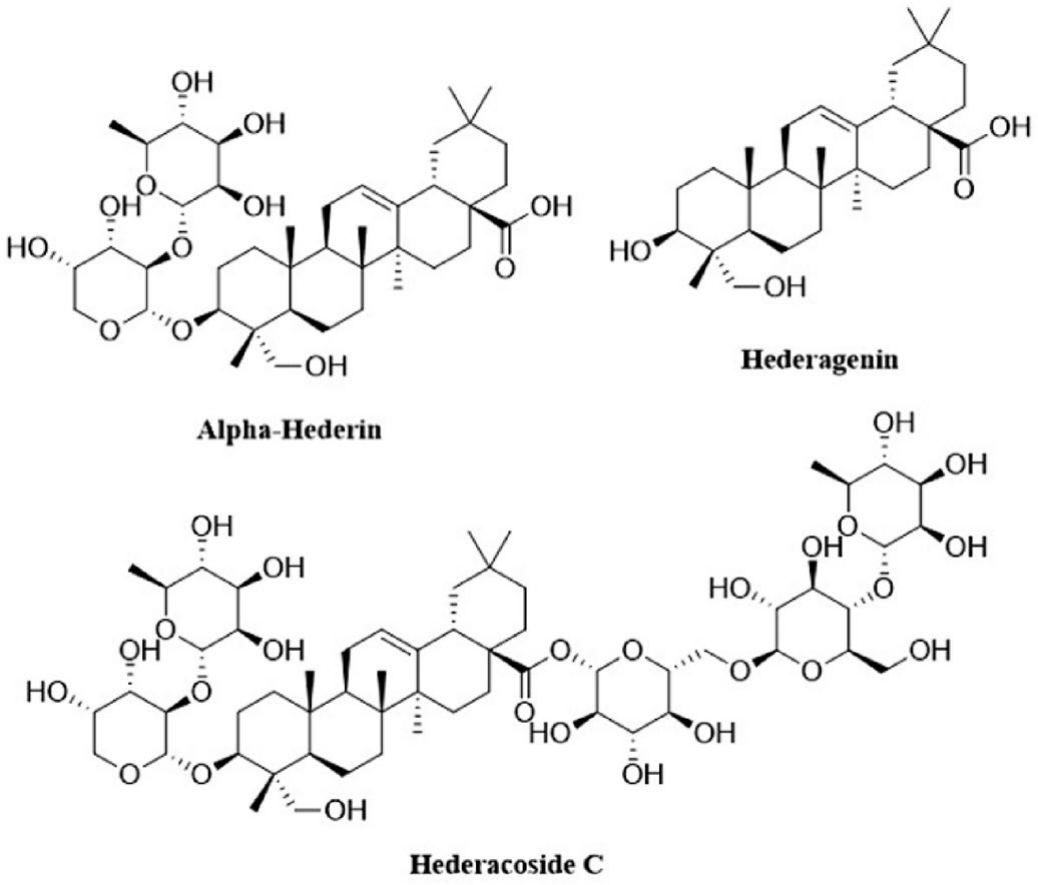
Chemical structures of the main triterpenoid saponins from *H. pastuchovii*.

The genus *Hedera* L. (Araliaceae family) comprises approximately 15 species distributed from Asia to North Africa and Europe^12^. Triterpenoid saponins, phenolic compounds, polyacetylenes, and volatile oils constitute the primary chemical constituents of this genus^13,14^. α-Hederin, hederagenin, and hederacoside C (Fig. 1) are among the major saponins identified in this genus and have been extensively investigated for their pharmacological activities^15,16^. Traditionally, plants of the genus *Hedera* have been utilized in folk medicine for the treatment of respiratory, hepatic, and renal disorders, as well as wound healing and skin infections. Extracts and isolated compounds from this genus have demonstrated diverse biological activities, including antimicrobial, antiparasitic, anti-inflammatory, antidiabetic, and hepatoprotective effects. One of the most notable pharmacological properties of this genus is its anticancer activity, which is thought to be associated with its saponin constituents^13^.

Numerous studies have reported the anticancer properties of *H. helix*^17^, *H. nepalensis*^18^, and isolated saponins from the genus *Hedera*, such as α-hederin^19^, hederagenin^20^, and hederacolchiside-A1^21^, in various cancer cell lines. However, the anticancer properties of *H. pastuchovii* Woronow have not been investigated to date.

*Hedera pastuchovii* Woronow is a plant native to Iran, Iraq, the North Caucasus, and Transcaucasus regions^22^. Antioxidant and antibacterial activities of the ethanolic extract of this plant have been previously reported^23^. The presence of oligosaccharides^24^, phenolic compounds, flavonoids^23^, sapogenins such as oleanolic acid and hederagenin^25^, as well as pastuchoside B (a triterpene glycoside structure identified)^26^, and pastuchoside C (another characterized triterpene glycoside), has been documented in its phytochemical profile^27^.

Based on previous studies that highlight the anticancer potential of the *Hedera* genus and considering the presence of bioactive compounds such as hederagenin in *H. pastuchovii* together with its high antioxidant capacity, this study aimed, for the first time, to investigate the cytotoxic and genotoxic effects of different solvent fractions from its leaf extract instead of the crude extract. Through MTT assay, flow cytometry, and micronucleus testing, their effects on lung (A549), breast (MCF-7), cervical (HeLa), and ovarian (SKOV3) cancer cell lines were evaluated, along with genoprotective effects in human lymphocytes, providing an initial exploratory understanding of their biological activity in cancer and normal cells.

## Materials and methods

### Chemicals and materials

Human cancer cell lines, including A549 (lung carcinoma), HeLa (cervical carcinoma), MCF-7 (breast adenocarcinoma), and SKOV3 (ovarian carcinoma), were supplied by the National Cell Bank of Iran (Pasteur Institute of Iran, Tehran, Iran). Ethanol (96%), *n-*hexane, ethyl acetate, methanol, silica gel, TLC sheets, sodium chloride, Folin-Ciocalteu’s reagent, sodium carbonate, aluminum chloride, potassium acetate, potassium chloride, diethyl ether, *n-*butanol, MTT, and dimethyl sulfoxide (DMSO) were purchased from Merck (Germany). Normal saline was purchased from Samen Pharmaceutical Co. (Iran). Gallic acid, quercetin, phosphate-buffered saline (PBS), trypsin, ethylenediaminetetraacetic acid (EDTA), annexin V, propidium iodide (PI), phytohemagglutinin (PHA), and cytochalasin B were supplied by Sigma-Aldrich (USA). Dulbecco’s Modified Eagle’s medium (DMEM), Dulbecco’s Modified Eagle Medium/Nutrient Mixture F-12 (DMEM/F12), Eagle’s Minimum Essential Medium (EMEM), fetal bovine serum (FBS), and RPMI 1640 Medium were purchased from GIBCO (USA). Penicillin and streptomycin were purchased from Jaber Ebne Hayyan Pharmaceutical Co. (Iran). Cell incubation was carried out using a CO_2_ incubator (Binder, Germany). Cisplatin was obtained from Sobhan Oncology Co. (Iran).

### Collection and total extraction

Leaves of *H. pastuchovii* Woronow were collected from the Caspian Hyrcanian forests (Chalmardi, Hezar-jerib, Neka; 36° 33′ 42″ N 53° 23′ 31″ E), Mazandaran, Iran. The collection was conducted during scientific field expeditions under the direct authorization and supervision of Dr. Masoud Azadbakht (PhD of Botany and Plant Science, Department of Plant Systematics, High Educational of Sanna Institute, Sari, Iran). The plant material was also formally identified by Dr. Azadbakht, and a voucher specimen (no. E1-18-123) has been retained at the MAZUMS herbarium. The authors confirm that *H. pastuchovii* is not classified as an endangered or protected plant species in Iran. Therefore, no specific national or environmental licenses were legally required for its collection for academic research. However, the collection process was carried out with high precision and in strict accordance with institutional and national guidelines, ensuring that no harm was caused to the local environment or ecosystem. Furthermore, the entire experimental protocol, including the plant collection process, was reviewed and approved by the Ethical Committee of the Mazandaran University of Medical Sciences (Ethical Code: IR.MAZUMS.REC.1394.2156). After air drying and fine grinding, the powder (500 g) was macerated three times in 70% ethanol (5 L) for 48 h, followed by concentration with a rotary evaporator and lyophilization at − 45 °C for three days to yield a fine powdered extract^28^. This process ensured reproducibility and preservation of thermolabile compounds.

### Fractionation by vacuum liquid chromatography

**Fig. 2:**
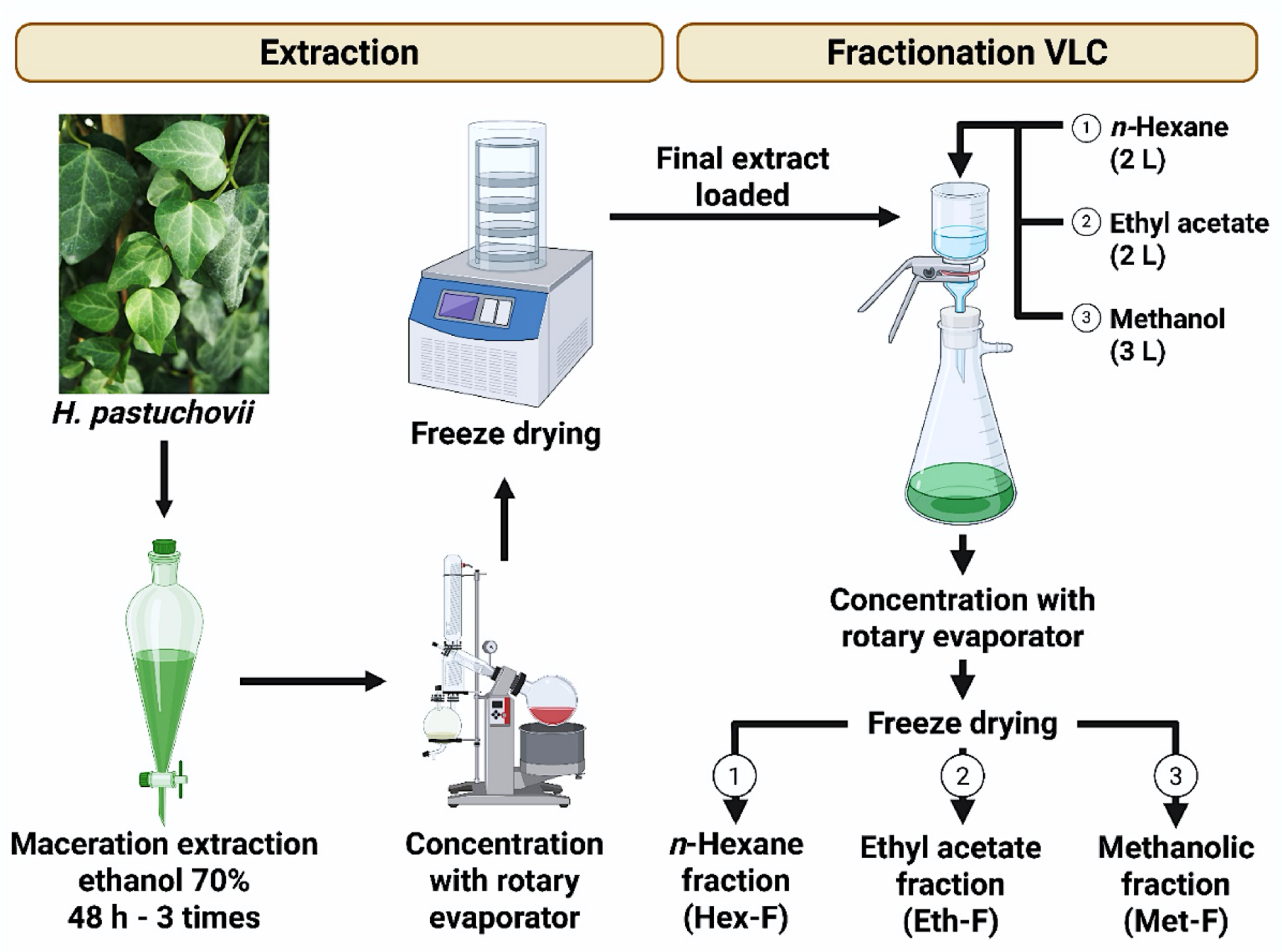
The extraction and fractionation procedure for *H. pastuchovii* leaves.

The ethanolic extract was fractionated by vacuum liquid chromatography (VLC) on silica gel (5 × 20 cm), sequentially eluted with *n*-hexane (2 L), ethyl acetate (2 L), and methanol (3 L). Each fraction was concentrated under reduced pressure using a rotary evaporator and then freeze-dried to yield *n*-hexane (Hex-F), ethyl acetate (Eth-F), and methanolic (Met-F) fractions in powdered form^29^ (Fig. 2). This polarity‑based separation yielded distinct classes of phytochemicals.

### Phytochemical analysis

#### Qualitative and quantitative analysis of saponins

The presence of saponins was first confirmed using the foam test. In this test, ethanolic extract (50 mg) was mixed with distilled water (5 mL) and sonicated at 40 °C for 5 min. After cooling to room temperature for 20 min, the mixture was shaken vigorously for 20 s. The formation of a stable foam layer that persisted for 10 min indicated the presence of saponins^30,31^.

The hemolytic activity was then assessed. For this assay, normal saline (3 mL) was placed in two test tubes, followed by distilled water (0.5 mL; control) or ethanolic extract (1000 µg/mL; test). Two drops of venous blood from a healthy donor (informed consent obtained, Ethical Code: IR.MAZUMS.REC.1394.2156) were added to each tube, gently mixed, and incubated for 1 h. A drop from each tube was examined microscopically at × 40 magnification, with red blood cell hemolysis confirming the presence of saponins^32^. 

The total saponin content of *H. pastuchovii* leaves was measured using a modified Gelgelo method^33^. The dried plant material (5 g) was extracted twice with 20% ethanol (50 mL) at 55 °C for 90 min, followed by filtration and three successive extractions with diethyl ether (100 mL). After each extraction, the diethyl ether phase was carefully decanted and discarded. The aqueous phase was subsequently extracted three times with *n*-butanol (100 mL), and the retained *n-*butanol phase was washed with 5% sodium chloride (100 mL), concentrated, and dried under airflow. The total saponin yield (%) was calculated as:

$${\mathrm{Yield}} = {\text{Weight of collected precipitate}}/{\text{weight of initial dried plant powder}} \times {1}00.$$

This gravimetric approach provided a reliable estimate of crude saponin content.

#### Determination of total triterpenoid content

The total triterpenoid content (TTC) of the fractions was determined using the vanillin-glacial acetic acid colorimetric method, following the procedure described in the literature with minor modifications. In brief, the sample solution (0.5 mL) was mixed with 5% vanillin (5 mL; w/v) in glacial acetic acid and perchloric acid. The reaction mixture was heated at 60 °C for 15 min, quickly cooled on ice, and subsequently diluted with glacial acetic acid (25 mL). The absorbance was recorded at 548 nm against a reagent blank using a UV–Vis spectrophotometer. A standard calibration curve was prepared with ursolic acid, and the TTC was expressed as milligrams of ursolic acid equivalents (UAE) per gram of each dried fraction^34^.

#### Determination of total phenolic content

The total phenolic content of the fractions was determined using the Folin-Ciocalteu method. Briefly, 0.5 mL of extract (400 µg/mL) was mixed with 0.2 N Folin-Ciocalteu reagent (2.5 mL) and stirred for 5 min, followed by the addition of sodium carbonate solution (2 mL; 75 g/L). After a 2 h incubation at room temperature, the absorbance was measured at 760 nm using a UV–visible spectrophotometer. Results were calculated from a gallic acid calibration curve and expressed as milligrams of gallic acid equivalents per gram of extract (mg GAE/g extract)^35,36^.

#### Determination of total flavonoid content

The total flavonoid content of the fractions was determined by a colorimetric method. An extract solution (400 µg/mL) was prepared and mixed with methanol (1.5 mL), 10% aluminum chloride (0.1 mL), 1 M potassium acetate (0.1 mL), and distilled water (2.8 mL). After incubation at room temperature for 30 min, the absorbance was measured at 415 nm using a UV–visible spectrophotometer. Quercetin was used as the standard for the calibration curve, and results were expressed as milligrams of quercetin equivalents per gram of extract (mg QE/g extract)^37^.

### Cell culture

All cell lines were authenticated by the provider (National Cell Bank of Iran, Pasteur Institute) before distribution. Cell lines were cultured in DMEM/F12 (A549), EMEM (HeLa), or DMEM (MCF-7 and SKOV3) supplemented with 10% FBS and 1% antibiotics and incubated at 37 °C in a humidified 5% CO_2_ atmosphere. Cells were subcultured into new plates when 70–80% confluency was reached^38,39^. All cell lines were maintained within a restricted low‑passage range (passages 3–10) in all experimental runs to preserve phenotypic stability and ensure data reproducibility. Cultures were routinely monitored for viability and regularly screened for contamination, with all samples remaining negative throughout the study.

### Cell viability test (MTT assay)

Cell viability was assessed using the MTT assay, which measures the reduction of MTT to formazan by mitochondrial enzymes in viable cells. A549, MCF-7, HeLa, and SKOV3 cells were seeded at 1 × 10^4^ cells/well in 96-well plates and incubated for 24 h in 100 µL of appropriate culture medium at 37 °C in a humidified atmosphere containing 5% CO_2_. Stock solutions of all fractions were prepared in DMSO and subsequently diluted with the respective culture media. In the highest treatment group (1000 µg/mL), the final DMSO concentration was 0.5% (v/v). Cells were then treated with different concentrations (10, 50, 100, 500, and 1000 µg/mL) of the Hex-F, Eth-F, and Met-F, or with cisplatin (3 µg/mL, used as a reference cytotoxic agent) as a positive control, for 48 h. Control wells received culture medium and DMSO (0.5% v/v). After treatment, MTT solution (20 µL; 5 mg/mL) was added to each well and incubated for 4 h at 37 °C in the dark. The medium was then removed, and DMSO (50 µL) was added to dissolve formazan crystals. The plates were gently shaken until the crystals completely dissolved, and absorbance was measured at 490 nm and 630 nm using a microplate reader. Cell viability was expressed as the percentage of surviving cells relative to the control^39,40^. Experiments were conducted using three independent biological replicates (n = 3), with three technical replicates per condition in each run to ensure reproducibility.

### Flow cytometric analysis of cellular apoptosis

Fractions with an IC_50_ below 1000 µg/mL in the MTT assay were selected for further experiments. Briefly, 2 × 10^5^ cells per well were cultured, and after incubation with or without extract (1000 µg/mL), the supernatant was removed, and the cells were washed with PBS (10 µL). Cells were detached for approximately 3 min using 0.25% trypsin and 0.1% EDTA, neutralized with medium (1 mL) containing 10% serum. The resulting cell suspension was combined with the previously removed medium and centrifuged at 1200 rpm for 5 min. The supernatant was discarded, and the cells were washed with PBS and centrifuged. Subsequently, cells were resuspended in 1% binding buffer (500 µL), centrifuged, and transferred to flow cytometry tubes. Annexin V antibody (2 µL) and propidium iodide (10 µL) were added, and samples were incubated at 37 °C in the dark for 30 min. Flow cytometry analysis was performed, with Q1–Q4 quadrants representing necrotic, early apoptotic, late apoptotic, and viable cells. The percentages of cells in each quadrant were determined using flow cytometry software^39^. Data were analyzed from at least 10,000 events per sample to ensure statistical reliability.

### In vitro micronucleus test for genotoxicity

Fractions with an IC_50_ below 1000 µg/mL in the MTT assay were selected for genotoxicity testing. Under strict sterile conditions, 5 mL of peripheral venous blood was collected from three healthy, non-smoking, non-alcohol-consuming volunteers with no recent antibiotic use or radiographic exposure. Informed consent was obtained from all participants prior to blood collection. Blood samples (1 mL aliquots) were cultured in RPMI medium (with 10% FBS and 1% antibiotics) to a final volume of 5 mL and incubated at 37 °C for 24 h. Lymphocyte proliferation was stimulated with PHA (0.1 mL), and cells were treated with fractions at 100, 500, and 1000 µg/mL and cisplatin (3 µg/mL), followed by a further 24 h incubation. The negative control was treated with normal saline, while the positive control was exposed only to cisplatin (3 µg/mL). Cytochalasin B (6 µg/mL) was then added to induce nuclear division.

After 72 h, cells were harvested, transferred to centrifuge tubes, and centrifuged at 800 rpm for 8 min. Following supernatant removal, a hypotonic potassium chloride solution (6 mL) was added and gently mixed via pipetting. Cells were immediately centrifuged at 1000 rpm for 5 min, and the supernatant was carefully aspirated, leaving approximately 1 mL of medium containing the cell pellet undisturbed. Fixation was achieved by gradual addition (dropwise) of 2 mL chilled fixative, increasing the total volume to 9 mL with additional fixative solution, followed by centrifugation at 800 rpm for 8 min. This fixation step was repeated at least three times until a clear supernatant was achieved. After the final centrifugation, the supernatant was adjusted to leave 0.5 mL of a uniform cell suspension.

For optimal fixation, samples were refrigerated at 4 °C for 24 h, and drops were placed on glass slides and air-dried at room temperature for 2–3 days. Slides were stained with Giemsa and examined under × 40 magnification; at least 1000 binucleated cells per sample were scored for micronuclei. Micronucleus frequency was used as a marker of genotoxicity, with reduced frequency indicating lower genetic damage in the experimental groups^41^. All assays were performed on independent blood samples from three different donors, and each sample was evaluated in triplicate. Furthermore, the capacity of Met‑F to protect human genomic integrity against chemically induced damage was evaluated by quantifying its genoprotective efficacy relative to baseline chromosomal damage induced by cisplatin alone, which served as a reference for assessing the effectiveness of Met‑F co‑treatment in mitigating this toxicity. Relative genoprotection was quantified as the percentage reduction in micronucleus frequency by the following mathematical relationship:

$${\text{Percentage Reduction }}\left( {{\% }} \right){ = }\left( {\frac{{{\mathrm{MF}}_{{{\mathrm{Cisplatin}}}} - {\mathrm{MF}}_{{\text{Cisplatin + Met-F}}} }}{{{\mathrm{MF}}_{{{\mathrm{Cisplatin}}}} }}} \right) \times 100$$

MF_Cisplatin_ denotes the micronucleus frequency induced by cisplatin alone, while MF_Cisplatin+Met‑F_ refers to the micronucleus frequency in lymphocytes co‑treated with cisplatin and the methanolic fraction. The percentage reduction values presented in Table 2 represent the mean ± standard deviation (SD) calculated from three independent biological replicates.

### Statistical analysis

Results for cell viability (MTT assay) and in vitro micronucleus test are expressed as mean ± SD. The data were analyzed by one-way ANOVA followed by a Tukey’s post hoc test for multiple comparisons using GraphPad Prism Version 8 software. A *p*-value less than 0.05 was considered significant. IC_50_ values were determined by nonlinear regression analysis using dose–response curves generated in GraphPad Prism and are presented along with their 95% confidence intervals (CIs). Graphical outputs were inspected to confirm goodness of fit and consistency across replicates. Flow cytometric analysis was conducted using percentage values derived from a single, representative screening run, with a minimum of 10,000 events evaluated per sample.

## Results

### Qualitative and quantitative analysis of phytochemical constituents

Since saponins are the prominent compounds in the *Hedera* species genus, the formation and stability of foam in the foam test and hemolysis of red blood cells in the hemolysis assay were observed, thus confirming the presence of saponins in the 70% ethanolic extract based on both qualitative tests. These qualitative assays were conducted solely on the crude extract as an initial screening to confirm the presence of saponins in the plant matrix before fractionation. In the quantitative total saponin content assay, the total saponin yield was calculated to be 29.95%. Following fractionation, the specific distribution and concentration of triterpenoid saponins and other components in the resulting fractions were rigorously quantified, rather than repeating subjective qualitative tests.

Quantification of total phenolic, flavonoid, and triterpenoid contents was carried out, and the results were expressed as gallic acid equivalents (GAE), quercetin equivalents (QE), and ursolic acid equivalents (UAE), respectively (Table 1). The calibration curves were established as follows: total phenolics, y = 0.0076x + 0.0253 (R^2^ = 0.9998, gallic acid); total flavonoids, y = 0.004x + 0.077 (R^2^ = 0.998, quercetin); and total triterpenoids, y = 0.0029x + 0.0362 (R^2^ = 0.996, ursolic acid). All values are presented as mean ± SD (n = 3). These results confirm that Met-F contained the highest levels of phenolics and triterpenoids, while Eth-F was the richest in flavonoids.

**Table 1:** Total phenolic, flavonoid, and triterpenoid contents of *H. pastuchovii* leaf fractions.

Fraction	Total phenolics (mg GAE/g extract)	Total flavonoids (mg QE/g extract)	Total triterpenoids (mg UAE/g extract)
*n-*Hexane	4.16 ± 0.23	1.93 ± 0.16	13.83 ± 1.25
Ethyl acetate	39.26 ± 3.12	23.34 ± 1.08	66.37 ± 5.18
Methanol	77.93 ± 4.28	15.30 ± 1.88	138.62 ± 4.88

Data are expressed as mean ± SD (n = 3). *GAE* gallic acid equivalents, *QE* quercetin equivalents, *UAE* ursolic acid equivalents.

### The cytotoxicity of different H. pastuchovii fractions on MCF-7, HeLa, SKOV3, and A549 cancer cell lines

**Fig. 3:**
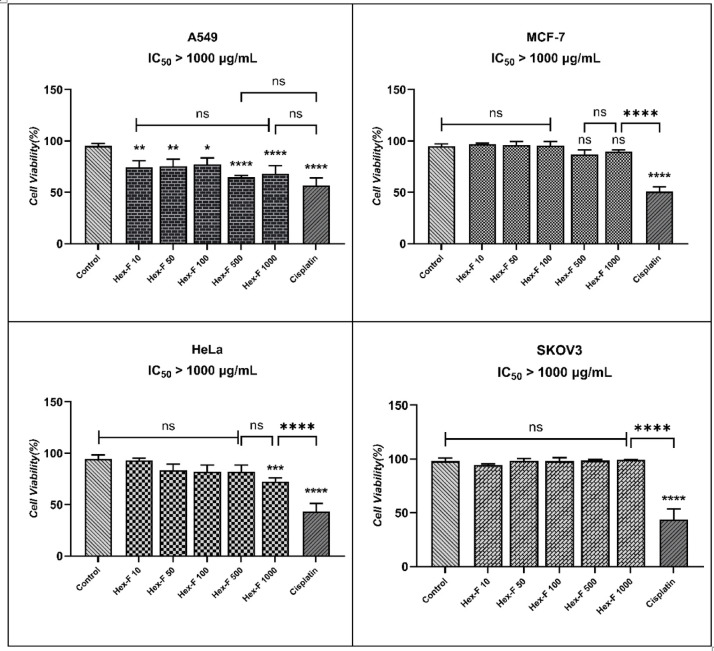
Cytotoxic effects of *H. pastuchovii n*-hexane fraction (Hex-F) on A549, MCF-7, HeLa, and SKOV3 cancer cell lines evaluated by the MTT assay. Data represent cell viability percentages compared to the control at different concentrations (µg/mL). Values are presented as mean ± SD (n = 3 independent biological replicates). Statistical significance compared to the control or between bracketed treatment groups is indicated by asterisks (ns: *p* > 0.05, *: *p* < 0.05, **: *p* < 0.01, ***: *p* < 0.001, ****: *p* < 0.0001).

**Fig. 4:**
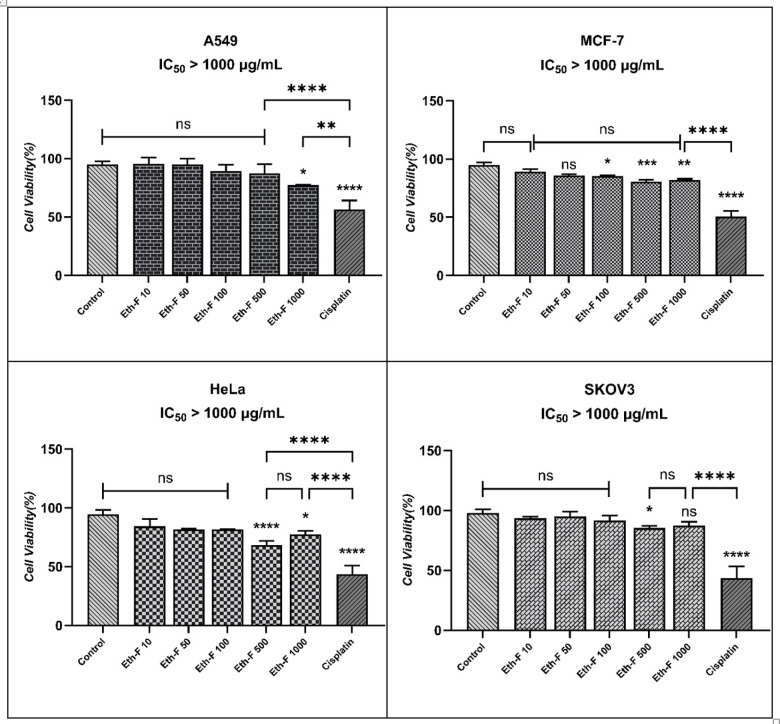
Cytotoxic effects of *H. pastuchovii* ethyl acetate fraction (Eth-F) on A549, MCF-7, HeLa, and SKOV3 cancer cell lines evaluated by the MTT assay. Data represent cell viability percentages compared to the control at different concentrations (µg/mL). Values are presented as mean ± SD (n = 3 independent biological replicates). Statistical significance compared to the control or between bracketed treatment groups is indicated by asterisks (ns: *p* > 0.05, *: *p* < 0.05, **: *p* < 0.01, ***: *p* < 0.001, ****: *p* < 0.0001).

**Fig. 5:**
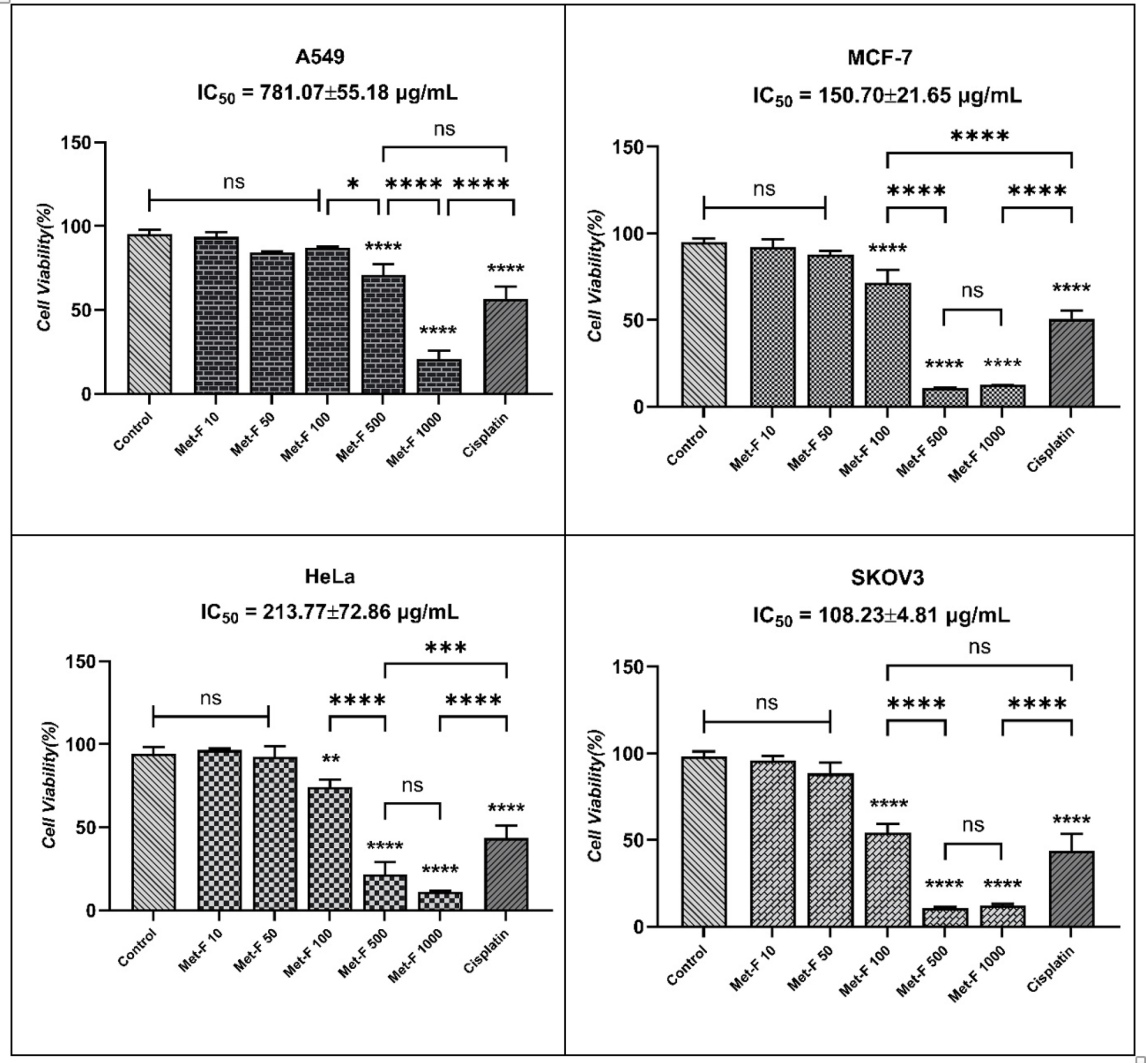
Cytotoxic effects of *H. pastuchovii* methanolic fraction (Met-F) on A549, MCF-7, HeLa, and SKOV3 cancer cell lines evaluated by the MTT assay. Data represent cell viability percentages compared to the control at different concentrations (µg/mL). Values are presented as mean ± SD (n = 3 independent biological replicates). Statistical significance compared to the control or between bracketed treatment groups is indicated by asterisks (ns: *p* > 0.05, *: *p* < 0.05, **: *p* < 0.01, ***: *p* < 0.001, ****: *p* < 0.0001).

The MTT assay results are shown in Figs. 3, 4, and 5. Hex‑F produced its strongest effect in A549 cells. Significant cytotoxicity was observed at all tested concentrations (10–1000 µg/mL, *p* < 0.05). The response was dose‑independent, with no significant differences between doses (*p* > 0.05). At 500 and 1000 µg/mL, Hex‑F exhibited a profound reduction in cell viability compared to the control group (*p* < 0.0001). In HeLa cells, Hex‑F showed no activity up to 500 µg/mL, but at 1000 µg/mL, viability decreased to 72.28 ± 3.96% (*p* < 0.001). No significant effects were detected in MCF‑7 or SKOV3 cells. IC_50_ values were not reached within the tested range (up to 1000 µg/mL).

Eth‑F demonstrated its highest activity in HeLa cells. No effect was detected below 100 µg/mL (*p* > 0.05). At 500 µg/mL, Eth‑F inhibited growth significantly (*p* < 0.0001). The effect at 1000 µg/mL was slightly weaker but not statistically different from 500 µg/mL (*p* > 0.05). This trend, most evident in HeLa cells, was also seen in MCF‑7 and SKOV3 cells, though differences were not significant. In MCF‑7 cells, Eth‑F showed no activity up to 50 µg/mL, but growth inhibition was observed at 100, 500, and 1000 µg/mL (*p* < 0.05). In SKOV3 cells, significant inhibition occurred only at 500 µg/mL (*p* < 0.05). In A549 cells, cytotoxicity was evident only at 1000 µg/mL (*p* < 0.05). IC_50_ values again exceeded 1000 µg/mL.

Met‑F exhibited the strongest activity across all tested cell lines. At 1000 µg/mL, Met‑F inhibited growth significantly (*p* < 0.0001), demonstrating profound cytotoxic effects. Although in MCF‑7 and SKOV3 cells, optimal inhibition was achieved at 500 µg/mL, with no further increase at higher doses. In HeLa and A549 cells, cytotoxicity increased significantly between 500 and 1000 µg/mL (*p* < 0.0001). At 100 µg/mL, Met‑F significantly reduced viability in MCF‑7 (*p* < 0.0001), HeLa (*p* < 0.01), and SKOV3 cells (*p* < 0.0001), but showed no effect in A549 cells (*p* > 0.05). No activity was observed below 50 µg/mL. Specifically, Met-F exhibited the lowest IC_50_ value against SKOV3 (108.23 ± 4.81 µg/mL, 95% CI 96.28–120.19 µg/mL), followed by MCF-7 (150.70 ± 21.65 µg/mL, 95% CI 96.92–204.48 µg/mL) and HeLa (213.77 ± 72.86 µg/mL, 95% CI 32.77–394.77 µg/mL), while demonstrating a moderate to weak growth inhibitory effect on A549 cells (IC_50_ = 781.07 ± 55.18 µg/mL, 95% CI 644.00–918.14 µg/mL). These findings indicate Met‑F as the most potent fraction, with a consistent cytotoxic profile across the tested cancer cell lines.

### Detection of apoptosis induced by Met-F in treated cell lines using flow cytometric analysis

**Fig. 6:**
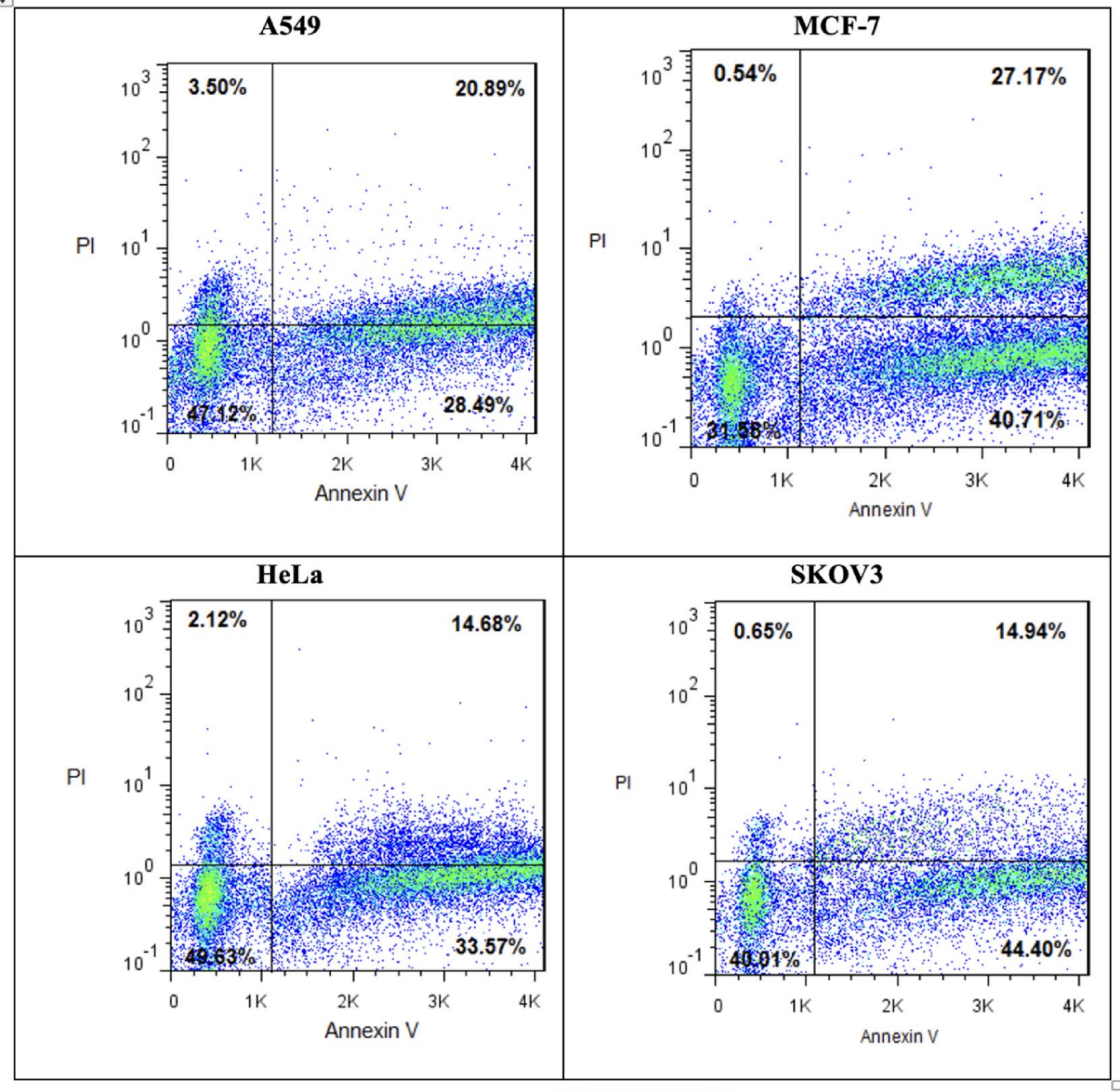
Flow cytometric analysis of apoptosis in cancer cell lines treated with *H. pastuchovii* methanolic fraction. Scatter plots and corresponding quadrant percentages are shown from a single representative screening run, with at least 10,000 events analyzed per sample.

The results of the flow cytometric analysis are presented in Fig. 6. The highest cell viability was observed in HeLa, A549, SKOV3, and MCF‑7 cell lines, with viability rates of 49.63%, 47.12%, 40.01%, and 31.58%, respectively. The greatest proportion of early apoptotic cells was detected in the SKOV3 cell line (44.40%), followed by MCF‑7 (40.71%), HeLa (33.57%), and A549 (28.49%). For late apoptosis, the highest rates were observed in MCF‑7 (27.17%), A549 (20.89%), HeLa (14.94%), and SKOV3 (14.68%). The most pronounced necrosis was found in A549 cells (3.5%), followed by HeLa (2.12%), SKOV3 (0.65%), and MCF‑7 (0.54%).

A549 results differed slightly from the MTT assay. While flow cytometry indicated relatively higher viability, the MTT assay revealed the lowest viability for this cell line. This divergence highlights the need for complementary assays to capture cytotoxic responses.

### Dose-dependent genoprotective effects of Met-F in the in vitro micronucleus assay

**Fig. 7:**
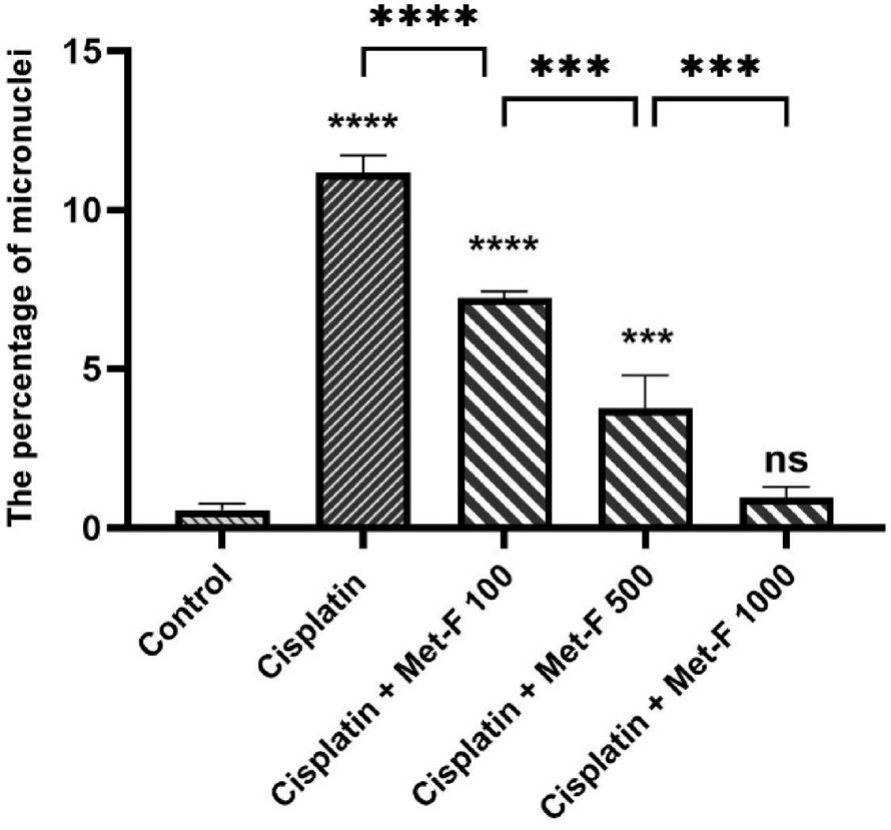
The percentage of micronuclei induced in vitro by cisplatin on cultured blood lymphocytes from human volunteers and protective effects of *H. pastuchovii* methanolic fraction (Met-F). Values are presented as mean ± SD (n = 3). Statistical significance compared to the control or between bracketed treatment groups is indicated by asterisks (ns: *p* > 0.05, ***: *p* < 0.001, ****: *p* < 0.0001).

The micronucleus assay results are shown in Table 2 and Fig. 7. Met‑F demonstrated a clear dose‑dependent genoprotective effect in cultured human lymphocytes. The strongest reduction in genotoxicity was observed at 1000 µg/mL, with values not significantly different from the control group (*p* > 0.05).

**Table 2:** The percentage of micronuclei induced in vitro by cisplatin on cultured blood lymphocytes from human volunteers and protective effects of *H. pastuchovii* methanolic fraction (Met-F).

Groups	Micronucleus frequency (mean ± SD)	% Reduction vs. Cisplatin
Control	0.57 ± 0.21	–
Cisplatin	11.17 ± 0.55****	–
Cisplatin + Met-F 100	7.23 ± 0.21****	35.14 ± 3.01
Cisplatin + Met-F 500	3.77 ± 1.02***	65.92 ± 10.55
Cisplatin + Met-F 1000	0.97 ± 0.32^ns^	91.37 ± 2.79

***: p 0.001

At 100 µg/mL, Met‑F reduced genotoxicity significantly compared with untreated cells (*p* < 0.05). At 500 µg/mL, protection increased further, and at 1000 µg/mL, micronucleus frequency decreased by up to 91.37 ± 2.79% relative to cisplatin‑treated lymphocytes. These results confirm that Met‑F not only lacks genotoxicity but also protects genomic integrity in a concentration‑dependent manner.

## Discussion

The objective of this study was to evaluate and compare the cytotoxic activity of different fractions derived from the leaf extract of *H. pastuchovii* against several cancer cell lines, aiming to explore its therapeutic potential. The findings provided insight into the possible anticancer properties of this plant and its active fractions.

The MTT assay showed that Met-F exhibited the highest cytotoxic activity against all four tested cancer cell lines: A549, MCF-7, HeLa, and SKOV3. In contrast, the less polar Hex-F and Eth-F exhibited only limited cytotoxic activity. The clear difference in activity raises the possibility that the major bioactive compounds of the plant are likely polar in nature, and the polarity-based fractionation strategy effectively concentrated and isolated anticancer compounds from *H. pastuchovii*. The phytochemical results provide a suggestive chemical basis for this interpretation, as the crude ethanolic extract contained a high proportion of polar constituents, mainly saponins (29.95%), and Met-F was particularly rich in phenolic compounds. However, in the absence of detailed chromatographic profiling and further in-depth mechanistic characterization, these findings serve only as preliminary hypotheses and tentative indicators of compound classes rather than providing definitive single-molecule identifications or fully established pathways. Furthermore, at 1000 µg/mL, the Met-F caused a marked reduction in cell viability. While a 1000 µg/mL concentration may appear elevated compared to pure single molecules, it represents the highest dose applied in this study. The pharmacological relevance of Met-F is more accurately reflected by its IC_50_ values. For the MCF-7 and SKOV3 cell lines, these IC_50_ values were substantially below the 200 µg/mL threshold (150.70 ± 21.65 and 108.23 ± 4.81 µg/mL, respectively). According to the National Cancer Institute protocols, cytotoxic compounds with IC_50_ values between 21 and 200 µg/mL are considered to have moderate activity^42,43^. This established criterion, together with the present outcomes, suggests that the polar fraction of *H. pastuchovii* demonstrates a valuable cytotoxic profile. As a result, these findings may suggest the presence of cytotoxic constituents within the polar matrix that, when concentrated, produce substantial inhibitory effects at this dose. This outcome underscores the preliminary relevance of this fraction for future isolation studies.

The results reveal a clear pattern when compared with earlier studies on other Hedera species. A saponin-rich fraction of *H. helix* demonstrated strong cytotoxicity against Hep-2 cells^44^. while a methanolic extract of *H. nepalensis* showed activity against the A549 lung carcinoma cell line with an IC_50_ of 28.4 µg/mL^45^. The purified saponin pulsatilla saponin A exhibited an IC_50_ of 2.80 µg/mL, confirming the potency of polar triterpenoid glycosides^45^. Furthermore, the methanolic extract of *H. nepalensis* also demonstrated potent cytotoxic potential^46^. Previous reports on *H. helix* also described cytotoxic effects against HeLa cervical carcinoma cells^17^, Ehrlich ascitic carcinoma cells^47^, breast cancer cells, including MCF-7 and MDA-MB-231^48,49^, and colon (HT-29) cancer cell lines^50^. The consistency of these findings across species supports the hypothesis that polar saponins and phenolics may contribute to anticancer activity observed in the genus *Hedera*. However, because individual pure compounds were not isolated or tested in the current work, this mechanistic link remains a literature-backed hypothesis that requires rigorous, compound-specific verification in future studies of *H. pastuchovii*.

Following the observation of Met-F’s cytotoxic activity, we investigated the mechanism underlying its effect on cancer cells. Annexin V/PI flow cytometric analysis demonstrated that Met-F predominantly induces programmed cell death through apoptosis. In all treated cell lines, a significant increase in the apoptotic cell population was observed, encompassing both early and late stages. Importantly, the percentage of necrotic cells was low across all groups, which supports the interpretation that Met-F triggers a controlled and non-inflammatory cell death mechanism. Consistent with these results, Elaidy et al. confirmed that α-hederin and hederagenin induce apoptosis by disrupting mitochondrial integrity, releasing cytochrome c, and activating caspase cascades^51^. In the case of the A549 cell line, a discrepancy between MTT and flow cytometry results was evident. The MTT assay indicated the lowest viability, while flow cytometry showed relatively higher survival. A similar divergence has been reported in studies where apoptosis and metabolic inhibition occurred at different time points^52^. Evidence also suggests that α-hederin and hederagenin can induce incomplete autophagy, which delays apoptosis and may explain the observed differences^19^. Despite the results obtained, due to the limited number of studies on *H. pastuchovii*, some of the points presented remain hypothetical, making further research necessary to precisely determine the mechanisms involved in apoptosis, identify contributing factors, and isolate the active compounds. Additionally, a limitation of this study is the assessment of cellular apoptosis by flow cytometry at only a single concentration (1000 µg/mL). As this investigation was intended as an exploratory screening, flow cytometry served exclusively as an endpoint qualitative assay to distinguish between apoptotic and necrotic pathways at the concentration yielding maximal cell death. However, since cell viability at this highest dose had ultimately decreased to approximately 50% in certain cell lines, constructing an accurate mechanistic dose–response curve would have necessitated even higher concentrations, which is not pharmacologically justifiable. Consequently, comprehensive mechanistic evaluations in this context will require further purification of compounds and fractions in future studies.

The micronucleus assay confirmed that Met‑F is non‑genotoxic and demonstrated a dose‑dependent genoprotective effect against cisplatin‑induced genomic damage in human lymphocytes. Based on the established literature, the genoprotective effect of Met-F is hypothesized to be mediated by phenolic and flavonoid constituents, which are recognized in other studies for their antioxidant capacity and ability to stabilize genomic integrity^17^. It should be noted that concurrent cancer‑directed cytotoxicity and protection of normal‑cell genomic integrity are highly significant in cancer therapy. Standard cytotoxic treatments often cause sub‑lethal genomic damage in healthy tissues, leading to therapy‑induced mutagenesis and long‑term secondary malignancies^53^. Furthermore, highly proliferative normal cells, especially peripheral lymphocytes, are particularly vulnerable, as conventional agents impair their proliferation and can cause severe immunosuppression and immune paralysis^54^.

The ability of Met‑F to protect the genomic integrity of human lymphocytes at therapeutic doses suggests a preliminary protective effect. By maintaining the genomic safety and proliferative balance of immune cells, such dual‑action therapies may help preserve immune competence, supporting immune‑mediated tumor clearance alongside direct antineoplastic effects. However, a key limitation of the present study is the lack of parallel cytotoxicity assays using non‑malignant human cell lines. Although the data indicate strong genomic safety in healthy lymphocytes and targeted cytotoxicity in cancer models, definitive conclusions about absolute selective cytotoxicity require further validation. Notably, valuable protective effects were reported for *H. helix* extracts, which were toxic to breast cancer cells but safe for normal breast epithelial cells^48,49,55^.

Furthermore, in vivo studies also confirmed that hederagenin exerted strong antitumor effects in the H22 hepatocarcinoma mouse model without damaging vital organs^56^. These multifaceted outcomes suggest the preliminary biological relevance of *H. pastuchovii* as a natural source with preliminary potential to decouple direct tumor inhibition from host systemic genotoxicity. Another limitation of this study is the lack of compound‑level chemical characterization. While the assays provided reliable estimates of total phenolic, flavonoid, and triterpenoid contents, they did not enable identification or quantification of individual molecules. Consequently, attributing the observed biological activities to specific saponins or phenolics remains speculative and literature-based.

Future research directions should include the isolation and structural characterization of novel compounds, such as pastuchoside B and C, which may contribute to the observed activity^57^. A second important direction should involve an in vivo validation of efficacy and safety, including detailed pharmacokinetic studies^58^. A third direction should examine advanced delivery systems, such as nanocarriers, which have been shown to enhance the selectivity and potency of extracts^48–50^.

## Conclusion

This study characterizes the cytotoxic and genoprotective properties of leaf fractions from *H. pastuchovii*, demonstrating that the polar Met‑F possesses the most pronounced in vitro antiproliferative activity across SKOV3, MCF‑7, HeLa, and A549 cancer cell lines. The higher concentration of total phenolic and triterpenoid constituents within this polar fraction is hypothesized to underlie its enhanced biological activity. Met‑F induced apoptosis as the predominant mode of cell death, with minimal necrotic involvement, indicating a controlled and regulated cytotoxic response. A notable outcome of this work is the dual‑action profile of Met‑F: while exerting dose‑dependent cytotoxicity in malignant cells, it also reduced cisplatin‑induced chromosomal damage in normal human lymphocytes, suggesting a degree of genomic protection under the tested conditions. This combination of antineoplastic and genome‑stabilizing effects highlights the preliminary biological relevance of the polar fraction as a source of bioactive compounds with complementary biological actions. These findings establish a clear foundation for future investigations, including bioassay‑guided isolation of active molecules, detailed chromatographic and structural characterization, and comprehensive in vivo studies to define pharmacokinetics, safety, and therapeutic index. Such work will be essential to determine the extent of translational relevance of *H. pastuchovii* and to clarify the contribution of individual constituents to its observed biological profile.

## Data Availability

All data generated and analyzed in this study are included within the manuscript. The datasets that remain available include the summary MTT viability tables, the flow‑cytometry quadrant percentage outputs, and the micronucleus frequency summary tables. Due to the age of the study, the original raw datasets (including plate‑reader files, FCS flow‑cytometry files, and microscopy image archives) are no longer accessible. All available processed data have been fully incorporated into the present manuscript.
